# Species-Specific Effects of Woody Litter on Seedling Emergence and Growth of Herbaceous Plants

**DOI:** 10.1371/journal.pone.0026505

**Published:** 2011-10-20

**Authors:** Kadri Koorem, Jodi N. Price, Mari Moora

**Affiliations:** Department of Botany, Institute of Ecology and Earth Sciences, University of Tartu, Tartu, Estonia; Duke University, United States of America

## Abstract

The effect of litter on seedling establishment can influence species richness in plant communities. The effect of litter depends on amount, and also on litter type, but relatively little is known about the species-specific effects of litter. We conducted a factorial greenhouse experiment to examine the effect of litter type, using two woody species that commonly co-occur in boreonemoral forest—evergreen spruce (*Picea abies*), deciduous hazel (*Corylus avellana*), and a mixture of the two species—and litter amount—shallow (4 mm), deep (12 mm) and leachate—on seedling emergence and biomass of three understorey species. The effect of litter amount on seedling emergence was highly dependent on litter type; while spruce needle litter had a significant negative effect that increased with depth, seedling emergence in the presence of hazel broadleaf litter did not differ from control pots containing no litter. Mixed litter of both species also had a negative effect on seedling emergence that was intermediate compared to the single-species treatments. Spruce litter had a marginally positive (shallow) or neutral effect (deep) on seedling biomass, while hazel and mixed litter treatments had significant positive effects on biomass that increased with depth. We found non-additive effects of litter mixtures on seedling biomass indicating that high quality hazel litter can reduce the negative effects of spruce. Hazel litter does not inhibit seedling emergence; it increases seedling growth, and creates better conditions for seedling growth in mixtures by reducing the suppressive effect of spruce litter, having a positive effect on understorey species richness.

## Introduction

It is well known that the forest overstorey has significant effects on herb layer cover, composition, and diversity (e.g. [1]–[6]). These effects occur through multiple interacting mechanisms, such as changes in light availability [7], [8], soil characteristics [9], soil pH [6], [10], water availability [5], and in particular through the effects of plant litter [11]. Plant litter can intercept light and rain, change the surface structure and act as a mechanical barrier for seeds, seedlings and shoots [12], [13]. Litter can also influence the chemical properties and pH of the soil, nutrient availability, and the diversity of fungi and other soil organisms [12], [14].

Germination and establishment are two key stages in plant community assembly [15] that are particularly sensitive to the presence of litter [12]. Generally, the effect of litter on seedling establishment is negative, and this negative effect increases with increasing amount (see reviews [12], [16]). The magnitude of the effect that plant litter has on vegetation has been compared to the impact of competition or predation [16]. Hence, patterns of litter accumulation can strongly affect community dynamics and litter plays a direct role in structuring plant communities [12], [16].

The effect of litter also depends on the vegetation variable considered [16]. Litter can inhibit emergence [16], [17] through alteration of the physical environment (e.g. reduced light availability), mechanical effects (e.g. barriers to seedling emergence), and changes to the chemical environment (e.g. soil pH, leaching of phytotoxins; [12]). However, litter can also modify environmental conditions to have positive effects on seedling growth by maintaining soil moisture, moderating soil temperature, providing nutrients during decomposition, and reducing inter-specific competition [12], [16], [18]. Hence, plant litter can have differential effects on plant performance at different life stages but relatively little is known about these effects in the same study system.

Plant species can exert strong control over community dynamics, and one mechanism is through the species-specific effects of litter. In a meta-analysis of 35 published studies, the effect of litter origin contributed most to the variability in the data [16]. Differential effects of plant litter can occur through differences in litter structure and/or litter quality. For example, grass and tree litter have differential effects on seedling establishment due to litter structure [19]. Litter quality refers to the amount of nutrients and secondary chemicals, in general high levels of nutrients lead to faster decomposition rates, whereas high levels of secondary chemicals and structural carbohydrates slow decomposition [20]. Several studies have found species richness is reduced with poor litter quality [4], [21].

Litter in natural habitats is rarely monospecific, but consists of a combination of different litter types resulting from the species composition of the community, and redistribution of litter through wind and water [12]. In a review of litter decomposition studies, Richards et al. [22] reported that in approximately half of litter mixtures studied decomposition rates of litter mixtures were higher than expected based on the rates observed in single-species litter. Further, the inclusion of broadleaf litter into needle litter can promote the decomposition of needle litter and dramatically increase soil microbial biomass [23]. However, less is known about the effects of litter mixtures on seedling establishment and inconsistent results have been found. In some cases, the effect of litter mixtures on seedling emergence was as expected based on the impacts of single-species litter and their contribution to the litter mixture (i.e. the biomass ratio hypothesis) [24], [25], but non additive effects have also been found [26].

In a boreonemoral forest, Koorem and Moora [27] found higher species richness and biomass below the sub-canopy deciduous shrub, common hazel (*Corylus avellana* L., hereafter hazel) compared to the dominant evergreen canopy tree, Norway spruce (*Picea abies* L., hereafter spruce). Depth of the litter layer was only environmental variable differing under the two woody species, with three times deeper litter beneath spruce compared to hazel [27]. Spruce has poor quality litter with low nutrient concentrations and high levels of secondary chemicals [28], which may also impact on seedling establishment. Hazel has also been found to have a positive effect on the abundance of species of high conservation value [4], on soil nutrients, and the activity of soil microbes [29], [30]. It has been suggested that the positive influence of hazel on understorey species richness is related to the effects of litter [27], [29], but this has never been tested experimentally.

In this paper, we address several possible mechanisms for the negative impact of spruce on understorey species to provide a mechanistic explanation to the pattern observed by Koorem and Moora [27]. Specifically, under controlled greenhouse conditions we examined the effect of litter amount (shallow, deep and leachate) and litter type (spruce, hazel and mixed) on seedling emergence and growth of three forest herbs. In particular, we address the following questions:

- Does the effect of litter depend on litter amount? If seedling establishment is mainly impeded by mechanical characteristics of litter, we expect reduced emergence and growth with increasing depth independent of litter type and no effect of leachate.
- Does the effect of litter depend on litter type? If litter type is a key mechanism influencing seedling emergence and growth we expect greater inhibitory effects of spruce needle litter and its leachate than broadleaf hazel litter, independent of litter depth.
- Does the effect of litter amount depend on litter type? Is the negative effect of spruce observed by Koorem and Moora [27] simply due to increased litter depth *per se* or does the effect of litter amount depend on litter type?
- Does litter affect seedling emergence and growth differently and is this affected by litter amount and type? We expect seedling emergence to be more negatively affected by litter than growth.

## Materials and Methods

### Study species

We selected three common herbaceous species that co-occur in the understorey of boreonemoral spruce forest with hazel understorey as response species: *Geum rivale* L. (hereafter *Geum*), *Prunella vulgaris* L. (hereafter *Prunella*) and *Hypericum perforatum* Crantz (hereafter *Hypericum*) [27], [31]. These species are all clonal perennial forbs. Mature seeds were collected from Tartu County, Estonia in summer 2008. Seeds were stored at room temperature and moved to a fridge at −5°C a month before the commencement of the experiment to mimic winter conditions. To test the germinability of collected seeds, 100 seeds were randomly selected from all species and germinated in a Petri dish. Percent germination was 78 for *Geum*, 81 for *Prunella* and 48 for *Hypericum*.

### Experimental design

**Figure 1:**
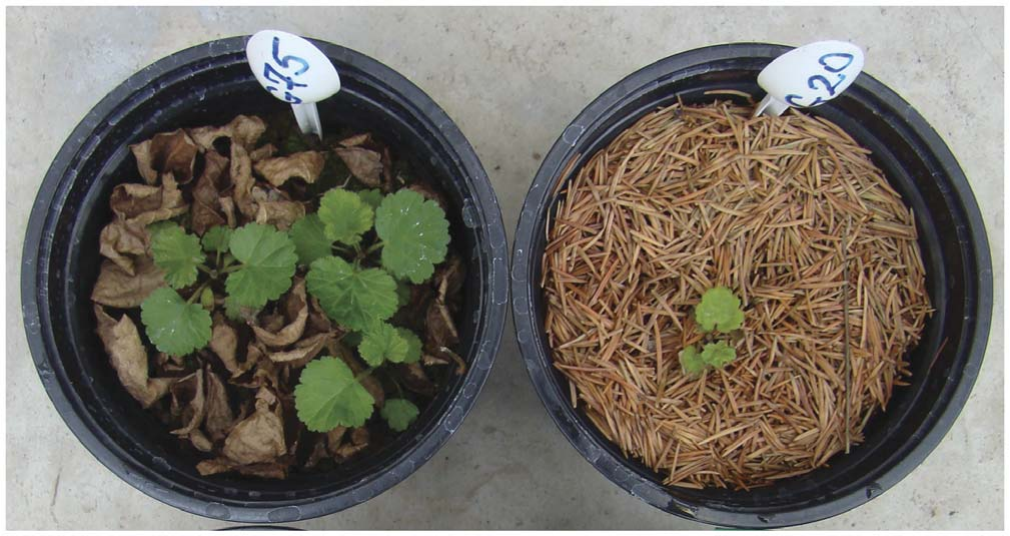
*Geum rivale* seedlings growing with deep hazel (left) and deep spruce litter (right).

Pots (1 dm^3^ volume, see Fig. 1) were prepared by mixing field soil and sand (ratio 4∶1). Soil was collected from a boreonemoral forest with relatively uniform soil conditions [32] to preserve the natural abundance and composition of soil organisms.

In the greenhouse, a fully factorial design (Table 1) was used to test the effect of litter amount (shallow, deep and leachate), litter type (spruce, hazel and spruce + hazel, hereafter mixed) on three understorey species (*Geum*, *Hypericum*, *Prunella*), giving 24 treatment combinations that were replicated 15 times. An additional 15 control pots were included for each plant species, which received no litter. Ten seeds were sown in each pot on the soil surface and were either covered with litter or left uncovered (controls).

**Table 1:** Number of pots with emerged seedlings (n for emergence analysis) and seedlings at the end of the experiment (n for biomass analysis) in the different litter treatments.

	Spruce			Hazel			Mixed			Control
	S	D	L	S	D	L	S	D	L	
**Emergence**										
*Geum*	15	8	13	15	15	15	15	15	15	15
*Hypericum*	13	2	15	14	15	15	12	8	15	15
*Prunella*	15	14	15	15	15	15	15	13	15	15
**Biomass**										
*Geum*	15	8	11	15	15	15	15	15	15	15
*Hypericum*	9	2	11	14	15	15	11	7	10	15
*Prunella*	15	14	15	15	15	15	15	13	15	15

Treatments: litter type (spruce, hazel, mixed, control-without litter addition), litter amount (S = shallow, D = deep, L = leachate) and understorey species (*Geum* = *Geum rivale*, *Hypericum = Hypericum perforatum*, *Prunella = Prunella vulgaris*).

Freshly senesced, undecomposed leaves of hazel were collected in autumn 2008 and stored at −20°C until use. Branches of spruce were cut in autumn 2008; fallen needles were collected afterwards and stored at −20°C. Litter of hazel was cut to smaller pieces (2 cm^2^) to provide an even coverage of the pots (Figure 1) and to enable litter mixtures to be formed. Cutting can change the physical structure of the litter and increased leaching or microbial degradation compared to field conditions, but as the leaves of deciduous hazel are fragile and decompose quickly [29], we do not expect this to significantly alter seedling responses in our experimental treatments.

Litter was applied to pots at 4 mm depth (hereafter shallow litter) and at 12 mm depth (hereafter deep litter), which was measured with a ruler in three places in each pot. For mixtures, equal amounts of spruce and hazel litter were mixed together and then applied to the pots. We were interested in the effect of depth and not mass, hence this differed for each species, mean weight for deep litter treatments was 16.42 g for spruce, 5.73 g for hazel and 11.72 g for mixed. The treatments simulate the mean depth of the litter layer found under hazel and spruce respectively [27]. Leachate was extracted by collecting 12 mm (same as the deep litter treatment) of spruce, hazel and mixed litter and placing it in mesh bags (15 bags, one per pot), which were kept in water (15×100 ml) for 48 h before the first application. Leachate (100 ml/pot) was then applied to pots every two days when the other pots received the same amount of tap water. Water was continually added to the mesh bags to simulate natural decomposition rates under field conditions. The leachate treatment was used to compare the chemical effects of spruce, hazel and mixed litter. The experiment commenced in February 2008 and ran for 65 days. Day length was 16 h of continuous light.

### Data collection

Seedlings were recorded as emerged once cotyledons were visible. After 26 days, we selected the three individuals that were most distant from each other and removed the others from the pot to avoid intraspecific competition. We recorded and removed emerged seedlings at regular intervals until the end of the experiment (65 days). The cumulative number of emerged seedlings was used in the analysis. Plants were removed from the pots and soil was washed away from the roots. Shoot and root biomass was harvested, dried at 70°C to a constant weight and weighed.

### Data analyses

We calculated logarithmic (log) response ratios to estimate seedling responses to the litter treatments [33], [34]. Log response ratio was calculated as: ln emergence or biomass of treated plant/average emergence or biomass of control plants. Log response ratios are standardized between all species, and therefore can be used to test for differences in the average species response to litter [34]. Seedling shoot and root mass was very small (especially for *Hypericum*), therefore total biomass per pot was used in further analyses. Total biomass of some plants was very small (<0.0001 g, the exact weight was not possible to measure with the scale used), therefore we added 0.0001 g to all biomass measures and those values were used for further analyses to able data of all seedlings to be used in the analysis. As the log response ratio can not be calculated for the pots without emerged seedlings, they were excluded from further analysis and therefore replication was reduced for some treatments (Table 1). Log response ratio may therefore overestimate emergence success; in current study, however, the results obtained with log response ratio are in accordance with mean emergence success (see Figure S1).

The effect of litter was considered significant when the 95% confidence of intervals did not overlap with zero (i.e. treated values were different from the control). We used three-way analysis of variance (ANOVA) with Tukey's HSD test to compare the log response ratio of seedling emergence and biomass of three understorey species under the different litter treatments (litter amounts: shallow, deep, leachate and litter types: spruce, hazel, mixed). ANOVA and Tukey HSD test were performed using Statistica (version 7.0, StatSoft Inc, Tulsa, U.S.A.).

This study complies with the laws of Estonia in which the greenhouse experiment was performed. No special permits were required.

## Results

### Seedling emergence

**Figure 2:**
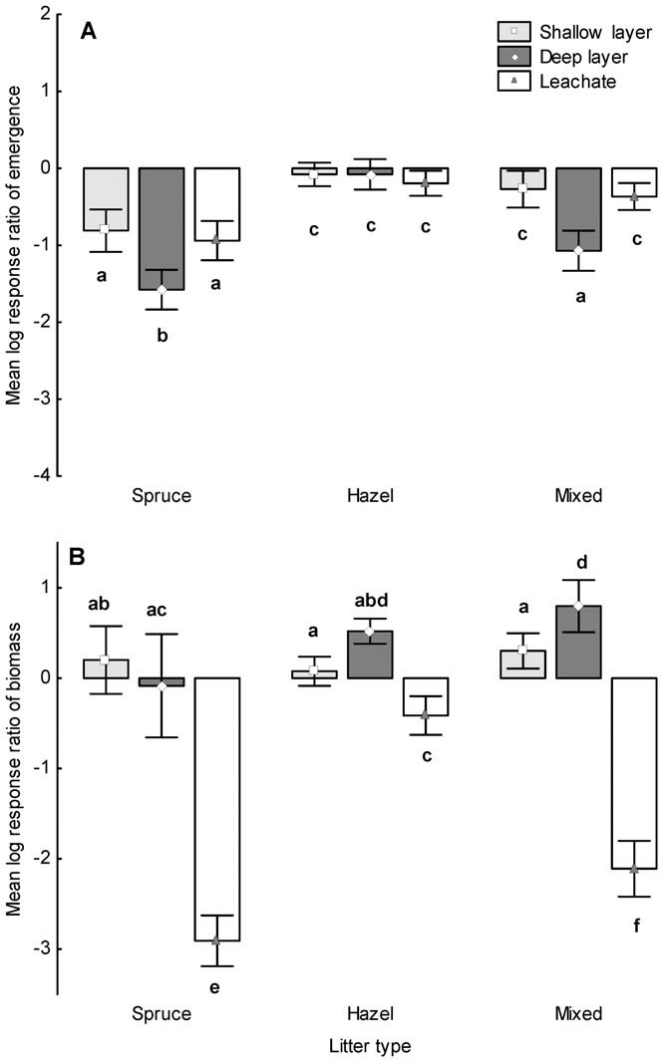
Mean (±95% confidence intervals) log response ratio of seedling emergence (A) and biomass (B) for the litter treatments. Letters indicate significant differences between treatments (P<0.05 Tukey HSD test).

Seedling emergence was significantly affected by the litter amount treatments (Table 2) with greater inhibition with deep litter (Figure 2A). Emergence was also significantly affected by litter type (Table 2), being lowest with spruce litter, intermediate with mixed litter and least suppressed by hazel litter (all three treatments differed significantly, Figure 2A). However, there was a significant interaction between litter amount and litter type (Table 2, Figure 2A). Spruce litter had a negative effect on emergence which increased with depth. Mixed litter also inhibited emergence, with the strongest negative effect in the deep litter treatment, whereas, seedling emergence in the shallow and deep hazel litter treatments did not significantly different from the control (95% confidence intervals overlapping 0-line), and there was no difference with increased depth (Table 2, Figure 2A).

**Table 2:** Results of three-way ANOVA on the effect of litter type (LT), litter amount (LA) and understorey species identity (S) on log response ratio of seedling emergence and biomass.

	Relative emergence				Relative biomass			
	d.f.	MS	F	P	d.f.	MS	F	P
**Variable**								
Intecept	1	128.01	456.1	<0.001	1	50.47	95.67	<0.001
Litter type (LT)	2	28.37	101.08	<0.001	2	25.18	47.73	<0.001
Litter amount (LA)	2	8.89	31.66	<0.001	2	171.3	324.69	<0.001
Species (S)	2	23.43	83,15	<0.001	2	6.51	12.34	<0.001
S × LT	4	1.29	4.6	0.001	4	3.12	5.92	<0.001
S × LA	4	1.44	5.13	<0.001	4	4.92	9.33	<0.001
LT × LA	4	3.59	12.78	<0.001	4	23.97	45.43	<0.001
S × LT × LA	8	0.22	0.79	0.61	8	3.22	6.1	<0.001
Error	337	0.28			324	0.53		

**Figure 3:**
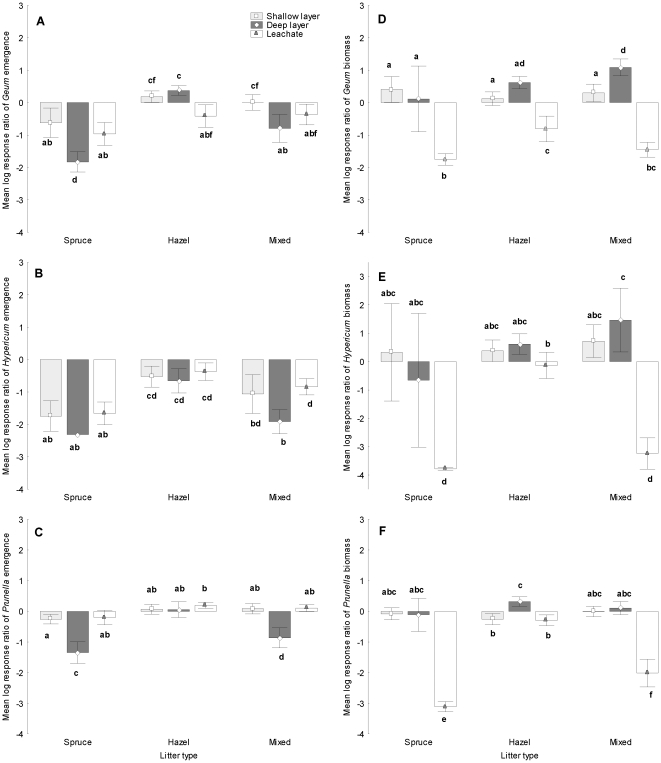
Mean (±95% confidence intervals) log response ratio of seedling emergence and biomass of *Geum* (A, D respectively), *Hypericum* (B, E) and *Prunella* (C, F) in the litter treatments. Letters indicate significant difference between treatments (P<0.05 Tukey HSD test).

There was also a significant interaction between understorey species and litter amount (Table 2, Figures 3A, 3B and 3C). Emergence of *Geum* was mildly suppressed by shallow litter, and inhibited more by deep litter (Figure 3A). Emergence of *Hypericum* was equally highly suppressed by shallow and deep litter (Figure 3B). Emergence of *Prunella* was suppressed only by deep litter, while shallow litter had no significant effect (Figure 3C). There was also a significant interaction between understorey species and litter type (Table 2, Figures 3A, 3B and 3C). Emergence of all species was strongly inhibited by spruce and less suppressed by mixed litter. The effect of hazel litter was different for all species: the effect was significantly negative for *Hypericum*, neutral for *Geum* and slightly, but significantly positive for *Prunella* (Figures 3A, 3B and 3C). The effect of litter differed significantly between all the litter types for emergence of *Geum* and *Hypericum* (Figures 3A and 3B), but there was no difference between hazel and mixed litter on the emergence of *Prunella* (Figure 3C).

Leachate significantly inhibited seedling emergence compared to the control (Figure 2A), with greatest inhibition with spruce leachate and significantly less inhibition in the hazel and mixed litter treatments which did not differ from each other (Table 2, Figure 2A). Both *Geum* and *Hypericum* responded similarly to leachate (Figures 3A and 3B), but *Prunella* had a positive response to hazel and mixed litter leachate and a slightly negative response to spruce leachate (Figure 3C).

### Seedling biomass

All litter amounts had a significant effect on biomass compared to the control (95% confidence intervals were not overlapping the 0-line), and differed significantly from each other, the positive effect of litter on biomass increased with depth (Table 2, Figure 2B). Compared to the control, biomass was also significantly affected by litter type and all three litter types differed significantly from each other (Table 2, Figure 2B). Spruce litter had a strong negative effect, mixed litter had a milder suppressive effect and hazel litter had a marginal, but significant positive effect on biomass. However, there was a significant interaction between litter amount and litter type (Table 2, Figure 2B). The effect of shallow and deep spruce litter did not differ from the control. Shallow hazel litter did not significantly affect biomass, but the effect was significantly positive for deep litter (Table 2, Figure 2B). Mixed litter had a significantly positive effect on biomass that increased with depth (Figure 2B).

There was also a significant three way interaction between litter amount, litter type and understorey species (Table 2, Figures 3D, 3E and 3F). For shallow litter, *Geum* and *Hypericum* had a neutral response to spruce and hazel litter and a positive response to mixed litter (Figures 3D and 3E). *Prunella* had a negative response to shallow hazel litter and no response to spruce and mixed litter (Figure 3F). For deep litter, all understorey species had a neutral response to spruce and a positive response to hazel litter, but the effect of mixed litter was positive for *Geum* and *Hypericum* and neutral for *Prunella* (Figures 3D, 3E and 3F).

Leachate had a significant negative effect on biomass compared to the control and all litter types differed significantly from each other (Table 2, Figure 2B). The greatest inhibition was found with spruce leachate, less with mixed litter and a mild negative effect of hazel leachate (Figure 2B). All understorey species responded significantly negatively to leachate of spruce and mixed litter, while leachate of hazel had a milder but also a significant negative effect on *Geum* and *Prunella*, and no impact on *Hypericum* (Figures 3D, 3E and 3F).

## Discussion

The aim of this study was to disentangle the effects of litter amount and type on understorey species to provide a mechanistic explanation to the increased species richness and biomass found under hazel compared to spruce [27]. Our experimental results confirm that both seedling emergence and biomass are strongly affected by litter amount (Table 2, Figures 2 and 3). As expected, increased depth of litter had a greater negative effect on seedling emergence suggesting a mechanical impediment to germination, consistent with other studies and providing some support to our first proposed mechanism (e.g. [16], [25], [35]), but the effect of litter amount depended on litter type supporting our third mechanism that neither litter amount or litter type alone can explain the patterns observed in the field. Increased depth of spruce litter had an increasing negative impact on seedling emergence, but this was not the case for hazel which had a neutral effect on emergence, regardless of depth.

The increased negative effect of spruce litter on seedling emergence with increased depth suggests the negative effect of spruce occurs partly through physical interference [12]. However, the effect of hazel litter was not different from control pots regardless of depth suggesting that the negative impact of spruce litter observed in the field is not due to depth *per se*. Different effect of litter types have been attributed to differences in litter structure [19], [25], [36], [37]. Donath and Eckstein [37] suggested that emergence from below oak litter may be easier compared to grass litter which forms dense mats on the ground, because seedlings may displace oak leaves during emergence. Spruce needle litter also forms dense mats, whereas hazel leaves stay loose on the ground and rapidly loose mass, and this might explain differential effects of litter type found here.

Chemical effects of litter are also important and are generally negative [12], but can also be positive depending on litter origin [24]. The negative effect of both hazel and spruce leachate on seedling emergence suggest in our case only inhibitory effects. Leachate of spruce had stronger negative effects on seedling emergence than hazel and mixed litter suggesting both mechanical and chemical inhibition of spruce on seedling emergence. Interestingly, hazel and mixed litter treatments did not differ from each other suggesting that in mixtures hazel reduces chemical inhibition by spruce. In mixtures, non-additive effects of leachate were found possibly because the rapidly decomposing hazel litter may dominate during the early stages of the experiment when seeds were germinating.

Seedling emergence of all three understorey species was significantly inhibited by spruce and mixed litter (Table 2, Figure 3). This is consistent with patterns in the field in which no herbaceous species were significantly associated with spruce [27]. Further, both *Hypericum* and *Geum* were found more frequently under hazel compared to spruce in the field [27]. Generally, seedling emergence was uniformly influenced by litter but there were some differences in responses to litter even among the similar species tested in this study. There are several reasons why species are differentially affected by litter, including seed size, germination cues and shoot morphology [17], [38]. It is not possible to determine the mechanisms operating in this study, but differences can be related to seed size. Seedling emergence under litter was lowest for *Hypericum* which have very small seeds (weight 0.008 g [39]) and was considerably higher for *Geum* and *Prunella* which have bigger seeds (weight 1.06 g and 0.8 g respectively [39]). A previous study found that *Hypericum* is suppressed by litter due to both the physical presence of litter (altering germination cues) and mechanical impediments probably due to small seed size [38].

Litter in natural habitats is rarely monospecific [12] and the litter experienced by understorey species in natural ecosystems is most similar to the mixed litter treatment. For seedling emergence, we found support for the biomass ratio hypothesis, with the effect of the litter mixtures intermediate between the single-species treatments [25]. Non-additive effects may be found if the inclusion of hazel litter increases spruce litter decomposition rates, but these effects might not be apparent in the current experiment for seedling emergence stage because decomposition rates might not have been affected. In our study, additive effects probably occur due to the changes in the litter structure in mixtures enabling greater seedling emergence compared with pure needle spruce litter.

The effect of litter on seedling growth ranged from neutral to positive contrasting with the mostly negative effects on seedling emergence. The positive effect of hazel litter that increased with depth on seedling growth may be due to more stable temperature and/or moisture conditions compared to bare ground [12], [19], [40] and/or faster decomposition rates of deciduous litter releasing nutrients for seedling growth [41]. The marginally positive effect of shallow spruce litter suggests that the litter structure does not create better conditions for growth compared to bare ground. It is also possible that positive and negative effects are balanced out, i.e. the positive effects are reduced by strong chemical inhibition resulting in neutral effects. Importantly, spruce litter does not inhibit growth, contrasting with its effects on seedling emergence. However, for biomass a strong negative effect of spruce extracts was found in both single-species and mixed litter treatments compared to a mild effect of hazel extracts. Hence, the physical presence of spruce litter is not negative for growth but chemical inhibition of growth still occurs.

Deep hazel and mixed litter had significant positive effects on seedling biomass that did not follow the biomass ratio hypothesis. The effect of mixing spruce litter with hazel creates better conditions for plant growth as suggested by the switch from a neutral effect of deep spruce litter to positive in mixtures. A probable mechanism for the positive influence of mixed litter on growth can be due to faster decomposition which has been reported for deciduous litter compared with evergreen litter [2], [29]. Accelerated litter decomposition releases nutrients more rapidly, turning litter mixtures into more favorable conditions for seedling growth. However, litter mixture effects on decomposition rates are difficult to predict from litter quality of species mixes [28], [42].

In conclusion, hazel has a positive influence on species richness in boreonemoral spruce forests [27] and this is partly due to plant litter – hazel litter does not inhibit seedling emergence, increases seedling growth and in mixtures creates better conditions for seedling growth by reducing the suppressive effect of spruce litter. The effects of litter differ according to type (structure and quality), amount, response species identity and life-history stage. Our study is the first that we know of reporting contrasting effects of litter mixtures at different life-history stages: additive effects for seedling emergence and non-additive effects for seedling biomass. However, as conditions in the glasshouse are relatively artificial compared to field conditions, the next step is to examine the effect of litter mixtures on different plant life-history stages under field conditions, incorporating natural microbial communities and different decomposition stages.

## Supporting Information

Figure S1 Mean emergence success (%± SE) of *Geum rivale* (A), *Hypericum perforatum* (B) and *Prunella vulgaris* (C) growing without litter (Control), with shallow layer (Sh), deep layer (De) and leachate (Le) of spruce, hazel and a mixture of spruce and hazel litter (Mixed).
(TIF)

## References

[1] Sydes C, Grime JP (1981). Effects of tree leaf litter on herbaceous vegetation in deciduous woodland. I. Field investigations.. J Ecol.

[2] Saetre P (1999). Spatial patterns of ground vegetation, soil microbial biomass and activity in a mixed spruce-birch stand.. Ecography.

[3] Augusto L, Dupouey JL, Ranger J (2003). Effects of tree species on understory vegetation and environmental conditions in temperate forests.. Ann Forest Sci.

[4] van Oijen D, Feijen M, Hommel P, den Ouden J, de Waal R (2005). Effects of tree species composition on within-forest distribution of understorey species.. Appl Veg Sci.

[5] Barbier S, Gosselin F, Balandier P (2008). Influence of tree species on understory vegetation diversity and mechanisms involved - A critical review for temperate and boreal forests.. Forest Ecol Manag.

[6] Wulf M, Naaf T (2009). Herb layer response to broadleaf tree species with different leaf litter quality and canopy structure in temperate forests.. J Veg Sci.

[7] Moora M, Daniell TJ, Kalle H, Liira J, Püssa K (2007). Spatial pattern and species richness of boreonemoral forest understorey and its determinants – a comparison of differently managed forests.. Forest Ecol Manag.

[8] Tinya F, Marialigeti S, Kiraly I, Nemeth B, Odor P (2009). The effect of light conditions on herbs, bryophytes and seedlings of temperate mixed forests in Őrség, Western Hungary.. Plant Ecol.

[9] Binkley D, Giardina C (1998). Why do tree species affect soils? The Warp and Woof of tree-soil interactions.. Biogeochemistry.

[10] Mölder A, Bernhardt-Romermann M, Schmidt W (2008). Herb-layer diversity in deciduous forests: raised by tree richness or beaten by beech?. Forest Ecol Manag.

[11] Janisova M, Hrivnak R, Gomory D, Ujhazy K, Valachovic M (2007). Changes in understorey vegetation after Norway spruce colonization of an abandoned grassland.. Ann Bot Fenn.

[12] Facelli JM, Pickett STA (1991). Plant litter: its dynamics and effects on plant community structure.. Bot Rev.

[13] Facelli JM, Pickett STA (1991). Plant litter - light interception and effects on an old-field plant community.. Ecology.

[14] Sayer EJ (2006). Using experimental manipulation to assess the roles of leaf litter in the functioning of forest ecosystems.. Biol Rev.

[15] Grubb PJ (1977). The maintenance of species-richness in plant communities: the importance of the regeneration niche.. Biol Rev.

[16] Xiong SJ, Nilsson C (1999). The effects of plant litter on vegetation: a meta-analysis.. J Ecol.

[17] Kostel-Hughes F, Young TP, Wehr JD (2005). Effects of leaf litter depth on the emergence and seedling growth of deciduous forest tree species in relation to seed size.. J Torrey Bot Soc.

[18] Jõgar Ü, Moora M (2008). Reintroduction of a rare plant (*Gladiolus imbricatus*) population to a river floodplain - How important is meadow management?. Rest Ecol.

[19] Donath TW, Eckstein RL (2010). Effects of bryophytes and grass litter on seedling emergence vary by vertical seed position and seed size.. Plant Ecol.

[20] Aerts R (1997). Climate, leaf litter chemistry and leaf litter decomposition in terrestrial ecosystems: a triangular relationship.. Oikos.

[21] Kooijman A, Cammeraat E (2010). Biological control of beech and hornbeam affects species richness via changes in the organic layer, pH and soil moisture characteristics.. Funct Ecol.

[22] Richards AE, Forrester DI, Bauhus J, Scherer-Lorenzen M (2010). The influence of mixed tree plantations on the nutrition of individual species: a review.. Tree Physiol.

[23] Li W, Pan KW, Wu N, Wang JC, Han CM, Liang XL (2009). Effects of mixing pine and broadleaved tree/shrub litter on decomposition and N dynamics in laboratory microcosms.. Ecol Res.

[24] Quested HM, Press MC, Callaghan TV (2003). Litter of the hemiparasite *Bartsia alpina* enhances plant growth: evidence for a functional role in nutrient cycling.. Oecologia.

[25] Quested HM, Eriksson O (2006). Litter species composition influences the performance of seedlings of grassland herbs.. Funct Ecol.

[26] Nilsson MC, Wardle DA, Dahlberg A (1999). Effects of plant litter species composition and diversity on the boreal forest plant-soil system.. Oikos.

[27] Koorem K, Moora M (2010). Positive association between understory species richness and a dominant shrub species (*Corylus avellana*) in a boreonemoral spruce forest.. Forest Ecol Manag.

[28] Jonsson M, Wardle DA (2008). Context dependency of litter-mixing effects on decomposition and nutrient release across a long-term chronosequence.. Oikos.

[29] Mohr D, Simon M, Topp W (2005). Stand composition affects soil quality in oak stands on reclaimed and natural sites.. Geoderma.

[30] Mohr D, Topp W (2005). Hazel improves soil quality of sloping oak stands in a German low mountain range.. Ann Forest Sci.

[31] Aavik T, Püssa K, Roosaluste E, Moora M (2009). Vegetation change in boreonemoral forest during post-logging succession – trends in species composition, richness and differentiation diversity.. Ann Bot Fenn.

[32] Zobel M, Kalamees R, Püssa K, Roosaluste E, Moora M (2007). Soil seed bank and vegetation in mixed coniferous forest stands with different disturbance regimes.. Forest Ecol Manag.

[33] Goldberg DE, Rajaniemi T, Gurevitch J, Stewart-Oaten A (1999). Empirical approaches to quantifying interaction intensity: competition and facilitation along productivity gradients.. Ecology.

[34] Vogt DR, Murrell DJ, Stoll P (2010). Testing spatial theories of plant coexistence: no consistent differences in intra- and interspecific interaction distances.. Am Nat.

[35] Ruprecht E, Jozsa J, Olvedi TB, Simon J (2010). Differential effects of several “litter” types on the germination of dry grassland species.. J Veg Sci.

[36] Sydes C, Grime JP (1981). Effects of tree leaf litter on herbaceous vegetation in the deciduous woodlands. II. An experimental investigation.. J Ecol.

[37] Donath TW, Eckstein RL (2008). Grass and oak litter exert different effects on seedling emergence of herbaceous perennials from grasslands and woodlands.. J Ecol.

[38] Bosy JL, Reader RJ (1995). Mechanism underlying the suppression of forb seedling emergence by grass (*Poa pratensis*) litter.. Funct Ecol.

[39] Royal Botanic Gardens Kew (2008). Seed Information Database (SID).. Version.

[40] Eckstein RL, Donath TW (2005). Interactions between litter and water availability affect seedling emergence in four familial pairs of floodplain species.. J Ecol.

[41] Cornwell WK, Cornelissen JHC, Amatangelo K, Dorrepaal E, Eviner VT (2008). Plant species traits are the predominant control on litter decomposition rates within biomes worldwide.. Ecol Lett.

[42] Hoorens B, Coomes P, Aerts R (2010). Neighbour identity hardly affects litter-mixture effects on decomposition rates of New Zealand forest species.. Oecologia.

